# Decoding Communication Difficulties in ADHD: A Narrative Review on the Potential Links with Dietary Patterns, the Microbiome, and Oxidative Stress

**DOI:** 10.3390/nu18162664

**Published:** 2026-08-14

**Authors:** Andreas Petropoulos, Pantelis Pergantis, Konstantinos Drosos, Dionysios Tafiadis

**Affiliations:** 1 Department of Physiotherapy, University of Thessaly, 35132 Lamia, Greece; 2 Net Media Lab & Mind & Brain R&D, Institute of Informatics & Telecommunications, National Centre of Scientific Research ‘Demokritos’, 15341 Agia Paraskevi, Greece; 3 School of Sciences, Speech and Language Pathology, European University Cyprus, 2404 Nicosia, Cyprus; k.drosos@euc.ac.cy; 4 Laboratory of Hearing, Speech, Communication Disorders and Mental Health, Department of Speech and Language Therapy, University of Ioannina, 45500 Ioannina, Greece

**Keywords:** attention-deficit/hyperactivity disorder, communication difficulties, gut microbiome, oxidative stress, nutrition

## Abstract

**Background/Objectives**: Individuals with attention-deficit/hyperactivity disorder (ADHD) often experience communication difficulties (CDs), including challenges with language development, pragmatic communication, and speech processing. While these difficulties are typically associated with cognitive and behavioral symptoms; emerging research suggests that biological and nutritional factors may also contribute. This narrative review aims to explore how diet patterns, gut microbiome changes and oxidative stress might be associated with communication difficulties in ADHD. **Methods**: This review was performed using the SANRA (Scale for the Assessment of Narrative Review Articles) framework. A structured/comprehensive literature search was performed across major databases including PubMed, Scopus and Web of Science. Google Scholar has been utilized as a complementary database to support emerging studies that have not yet been registered in these databases. The search focused on studies that investigated the connections between nutrition, microbiome dysregulation, oxidative stress, ADHD and communication-related outcomes. **Results**: Current research indicates that unbalanced diets, micronutrient deficiencies, and changes in the gut microbiome can lead to neuroinflammation and oxidative stress. All these biological processes may disrupt neurotransmission and neural connectivity, which can potentially affect brain regions and networks that are highly important for attention and language processing, including both cortical and subcortical structures. Therefore, communication difficulties in ADHD may, in part, stem from these interconnected neurobiological mechanisms. However, direct clinical evidence linking nutritional factors, gut microbiome alterations, or oxidative stress with communication-specific outcomes in ADHD remains very limited, as most available studies have focused on core ADHD symptoms, attention, executive functioning, or biological markers. **Conclusions**: Dietary factors, alterations in the gut microbiome, and oxidative stress may collectively contribute to the complex underlying causes of communication disorders in ADHD. This review proposes an integrative framework that links nutrition-related mechanisms to communication outcomes, emphasizing the potential impact of modifiable lifestyle factors. Further longitudinal studies are needed to establish causal relationships and determine the clinical effectiveness of targeted nutritional interventions for communication difficulties in ADHD.

## 1. Introduction

ADHD is one of the most overstudied neurodevelopmental disorders [1]. This condition affects 5–7% of children globally and is mostly defined by a set core of symptoms including inattention, hyperactivity and impulsivity [2]. All these can lead to severe impairment in day-to-day life, and their persistence varies from childhood to adulthood [3].

ADHD is also presented through various studies as a multifactorial disorder. Besides behavioral characteristics, it also involves executive function deficits impacting hot and cold executive processes that foster psychosocial and adaptive functioning [4]. Communication difficulties (CDs), on the other hand, seem to be a dimension of ADHD that remains underexplored [5]. These disorders have a significant impact on language processing and social competence to academic performance and can have a marked effect on emotional well-being and overall quality of life [6].

Apart from this, dietary patterns have also shed light to oxidative stress, and the gut microbiome might feed into the neurobiology of the disorder [7]. Imbalances caused by nutrition, like deficiencies in omega-3 fatty acids and key micronutrients, have been tied to neuroinflammatory pathways and alterations in neurotransmitter function in individuals with ADHD [8].

In the same way, gut microbiome dysbiosis may influence brain development and function through gut–brain axis signaling, thereby affecting cognitive, behavioral, and emotional regulation [9]. Oxidative stress has also been linked to neuronal dysfunction and neural connectivity, affecting brain networks involved in language, cognition, and communication [10].

Taken together, these findings support a multifactorial model in which diet-related, microbial, and biological pathways interact to influence not only core ADHD symptoms but also communication and neurodevelopmental outcomes [2]. Despite the growing body of evidence linking nutritional, microbial, and neurobiological mechanisms to ADHD, their potential contribution to communication difficulties in ADHD has not yet been systematically explored.

In this review, “communication difficulties” is used as a broad term covering reported difficulties in language, pragmatic communication, discourse organization, listening, speech processing, and social communication. The term “communication disorder” is used only when a formal clinical diagnosis is reported. “Communication-related functions” refers to cognitive, linguistic, auditory, or social processes that may support communication but do not themselves represent a diagnosed disorder. Communication difficulties associated with ADHD should also be distinguished from a co-occurring developmental language disorder. In some individuals, structural language difficulties meet the criteria for a separate clinical diagnosis, whereas in others, pragmatic, conversational, or listening difficulties may arise mainly from inattention, impulsivity, working-memory limitations, or executive dysfunction.

Therefore, this narrative review aims to integrate current evidence regarding dietary patterns, gut microbiome alterations, and oxidative stress in ADHD, with particular emphasis on their potential contribution to communication difficulties and disorders. By adopting an integrative and mechanistic perspective, this review seeks to provide a more comprehensive understanding of the pathways linking nutrition, neurobiology, and development to communication outcomes in ADHD.

## 2. Materials and Methods

A narrative review approach was selected as appropriate for this study as current evidence on nutritional and biological factors is emerging for communication difficulties in ADHD.

A comprehensive literature search was conducted using PubMed, Scopus, and Web of Science. Search term combined keywords and Medical Subject Headings (MeSH) related to ADHD, communication and language functions (e.g., “communication”, “language”, “speech”, and “pragmatics”), dietary factors (e.g., “diet”, “dietary patterns”, “micronutrients”, and “omega-3 fatty acids”), gut microbiome (e.g., “gut–brain axis” and “microbiome”), and oxidative stress.

The search syntax was adapted to the requirements of each database. The complete search strategies used for PubMed, Scopus, Web of Science, and Google Scholar are presented in Supplementary Table S4.

The initial literature search was conducted in May 2025 and was updated in June 2026 to identify recently published evidence. English-language publications available up to the date of the updated search were considered, with priority given to systematic reviews, meta-analyses, randomized controlled trials, and large observational studies. In addition, narrative reviews and studies exploring biological pathways were also included when they provided useful information about the relationships among nutrition, neurodevelopment, and communication.

Eligibility factors included the examination of associations between nutritional factors, gut microbiome alterations, oxidative stress and ADHD, highlighting neurocognitive and communication related outcomes. Studies focusing exclusively on unrelated conditions were generally excluded. However, selected studies from other clinical populations were retained when they provided relevant mechanistic information concerning biological processes that are not specific to a single diagnosis.

Given the heterogeneity of the available evidence and the limited number of studies directly investigating communication outcomes, a narrative synthesis approach was adopted. Studies assessing core ADHD symptoms, attention, executive functioning, or biological mechanisms without directly measuring communication were used to support the mechanistic discussion. They were not treated as direct evidence of an association with communication difficulties. The selected literature was reviewed and integrated to examine potential biological pathways linking nutrition, microbiome dysregulation, oxidative stress, and communication challenges in individuals with ADHD.

To ensure methodological rigor and reporting transparency, the present narrative review was developed and evaluated according to the Scale for the Assessment of Narrative Review Articles (SANRA), a validated instrument specifically designed to assess the quality of narrative review manuscripts. SANRA consists of six methodological domains: (i) justification of the article’s importance, (ii) statement of aims, (iii) description of the literature search, (iv) referencing, (v) scientific reasoning, and (vi) appropriate presentation of data. Each domain is scored using a three-point scale (0 = low standard, 1 = moderate standard, 2 = high standard), resulting in a maximum possible score of 12.

The final version of the manuscript was independently evaluated by two reviewers experienced in neurodevelopmental disorders and speech-language pathology. Both reviewers assessed each SANRA domain independently. Any discrepancies in scoring were resolved through discussion until full consensus was achieved. The final consensus assessment is presented in Supplementary Table S1.

Throughout the review process, particular emphasis was placed on ensuring a clearly defined clinical objective, a transparent literature search strategy, balanced and up-to-date referencing, critical synthesis of the available evidence, and logical organization of the scientific content. These principles guided both manuscript development and final quality assessment.

To enhance the methodological robustness of the narrative synthesis, the available literature was evaluated at the thematic level by considering the consistency of findings, the predominant study designs, and the directness of the evidence in relation to communication. Consistency was considered high when findings were broadly concordant, moderate when findings were generally supportive but heterogeneous, and emerging when evidence remained limited or primarily mechanistic. The overall level of support was judged according to the predominant study designs, the convergence of the available findings, and their directness in relation to the review question. Two reviewers completed the thematic appraisal independently, and disagreements were resolved through discussion and consensus. These ratings represent structured narrative judgments and do not constitute a formal risk-of-bias or GRADE assessment. Detailed summaries are provided in Supplementary Tables S2 and S3.

## 3. Communication Difficulties in ADHD: Phenotype and Neurobiological Mechanisms

### 3.1. Types of Communication Difficulties in ADHD

Communication difficulties (CDs) are more commonly recognized as a clinically significant feature of ADHD, affecting multiple domains of language, social communication, and interpersonal functioning [11]. These impairments extend beyond simple developmental delays and often involve complex deficits in expressive and receptive language, pragmatic communication, and verbal expression [12].

Reduced verbal fluency, impaired narrative organization, and difficulties in organizing and sequencing verbal information, and impairments in narrative coherence is frequently observed in children with ADHD [6]. Receptive language impairments in children with ADHD may involve difficulties understanding complex verbal instructions and reduced ability to process rapid or competing auditory input [13]. Pragmatic language impairments are particularly prominent in individuals with ADHD and may include inappropriate turn-taking, difficulties maintaining conversational coherence, and reduced sensitivity to social cues [14].

These communication impairments may also persist into adolescence and adulthood, contributing to academic underachievement, social difficulties, and reduced quality of life [15]. We emphasize that despite their clinical significance, communication difficulties in ADHD remain underrecognized and are frequently interpreted solely as secondary consequences of attentional dysfunction [16].

### 3.2. Neurocognitive Mechanisms Underlying Communication Impairments

Communication difficulties in ADHD are closely linked to underlying neurocognitive dysfunctions [17]. Deficits in working memory, inhibitory control, and cognitive flexibility significantly impact language processing and communicative competence [18]. For example, limitations in working memory can affect the ability to retain and manipulate linguistic information during conversations, while deficits in inhibitory control may result in impulsive, disorganized, or contextually inappropriate speech [19]. Furthermore, attentional dysregulation can affect the efficient processing of linguistic stimuli as well, especially in complex or rapidly changing communication environments [20].

### 3.3. Neural Substrates of Communication in ADHD

At the neurobiological level, communication impairments in ADHD have been associated with alteration in brain regions and neural networks involved in language processing and executive control [21]. Functional and structural abnormalities have been reported in the prefrontal cortex, which play a central role in executive functioning and discourse organization, as well as in temporal regions implicated in language comprehension and auditory processing [22].

In addition, atypical functioning of frontostriatal circuits may disrupt dopaminergic signaling, thereby affecting both cognitive control and speech production [23]. Emerging evidence also suggests the involvement of brainstem and subcortical auditory processing pathways, which may contribute to impairments in speech perception and auditory discrimination [24].

Altered connectivity among these neural systems may compromise the coordination of linguistic, cognitive, and sensory processes necessary for effective communication [25]. Together, these findings support the view that communication difficulties in ADHD arise from distributes network dysfunction rather than isolated cognitive or linguistic deficits [26].

Moreover, impairments in processing speed and temporal organization may disrupt the integration of auditory and linguistic information, thereby affecting both language comprehension and verbal production [27]. Collectively, these neurocognitive deficits contribute to the characteristic communication profile observed in individuals with ADHD. However, the available findings should be interpreted with caution. Communication profiles vary considerably across individuals with ADHD, and differences in age, symptom presentation, co-occurring developmental language disorders, medication status, and assessment methods may partly explain the variation across studies. In addition, many language and communication tasks place demands on attention, working memory, processing speed, and inhibitory control. It is therefore often difficult to determine whether poorer performance reflects a primary language difficulty, the effects of ADHD-related cognitive dysregulation, or a combination of both.

### 3.4. Linking Communication Difficulties with Environmental and Biological Pathways

While neurocognitive and neural mechanisms give a foundational explanation for communication impairments in ADHD, they may not fully reflect the heterogeneity observed in clinical presentation. Increasing evidence suggests that biological and environmental factors, including nutrition, gut microbiome dysbiosis, and oxidative stress, may modulate these underlying neurodevelopmental mechanisms [28,29].

These factors may influence neurodevelopmental processes, neurotransmitter systems, and neural connectivity, thereby affecting communication abilities [30]. Alterations in dopaminergic and serotonergic signaling, potentially driven by nutritional deficiencies or gut microbiome dysbiosis, may exacerbate executive and language-related dysfunction [31].

This perspective highlights the need to consider communication difficulties in ADHD within a broader biopsychological framework, in which nutritional, microbial, and biological pathways interact with cognitive and neural systems [32].

### 3.5. Linking Communication Difficulties with Nutritional and Biological Pathways

Current evidence suggests that CDs in ADHD may be influenced by a complex interplay between neurocognitive dysfunction and modifiable biological factors including dietary patterns, gut dysbiosis, neuroinflammation and oxidative stress [7]. These factors may act as upstream modulators shaping brain function and, as a consequence, communication outcomes [33].

**Figure 1:**
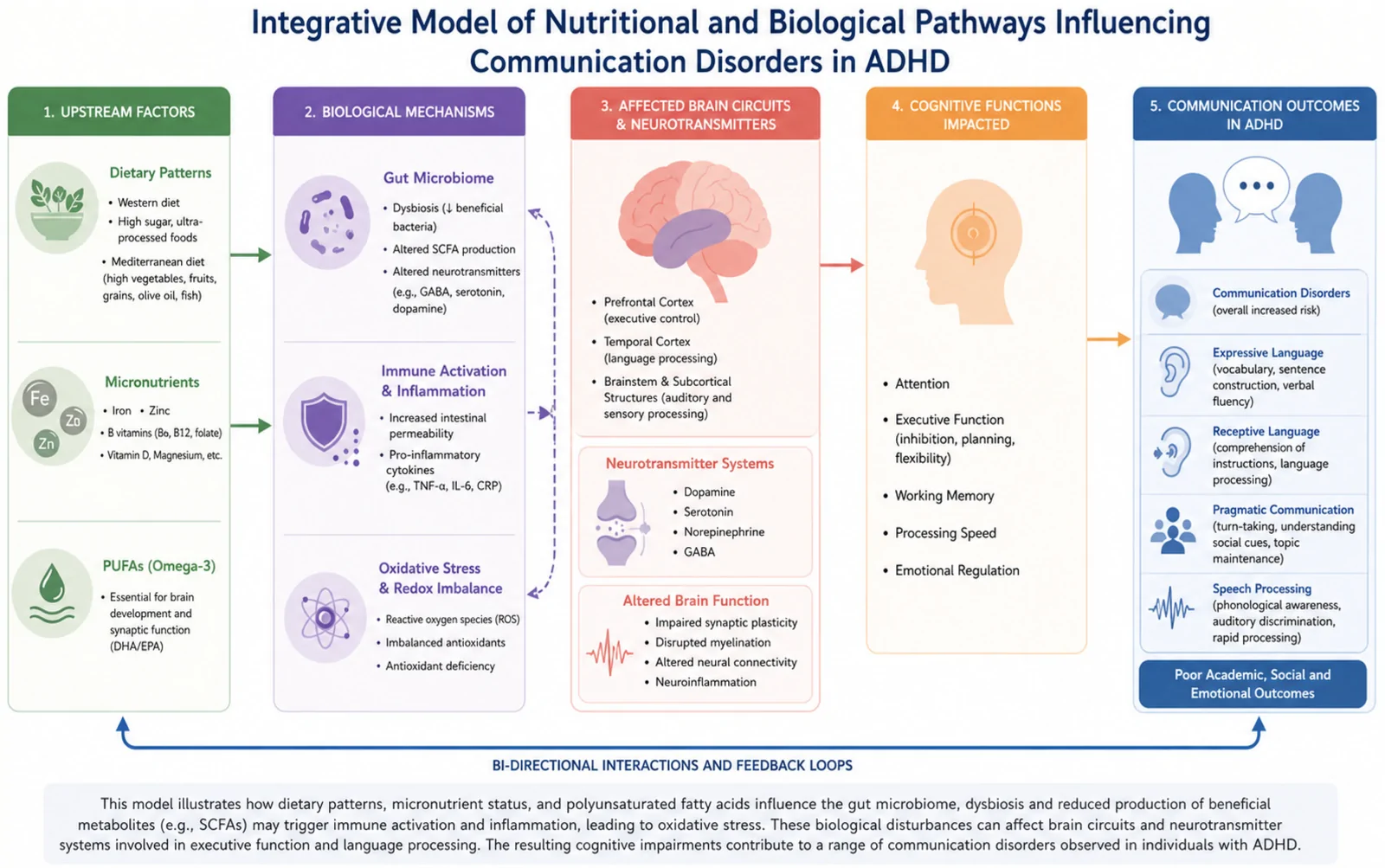
Integrative model of nutritional and biological pathways influencing communication difficulties in ADHD.

This integrative perspective provides the conceptual foundation for the subsequent section of this review, which examine how dietary patterns, micronutrients, gut microbiome alterations, and oxidative stress may contribute to communication challenges in ADHD (Figure 1). These proposed links remain indirect, as studies have not yet established that nutritional or microbiome alterations directly affect speech or language outcomes in individuals with ADHD.

## 4. Dietary Patterns and Neurocognitive Function in ADHD

### 4.1. Western Dietary Patterns and ADHD Symptomatology

Dietary patterns characterized by high consumption of ultra-processed foods, refined sugars, and saturated fats, commonly referred as Western diets, have been associated with increased risk and greater severity of ADHD symptoms [34]. These dietary habits are frequently accompanied by low intake of fiber, essential micronutrients, and bioactive compounds that are critical for optimal neurodevelopment and brain function [35].

Several observational studies have reported positive associations between Western dietary patterns and increased levels of inattention, impulsivity, and hyperactivity [36]. These effects may be mediated through multiple biological mechanisms, including systemic inflammation, altered glucose metabolism, and dysregulation of neurotransmitter systems, particularly dopaminergic signaling [7]. In addition, high glycemic loads and frequent consumption of simple sugars may contribute to rapid fluctuations in blood glucose levels, potentially leading to behavioral instability and impaired cognitive control [37].

From a neurocognitive perspective, these dietary patterns may negatively affect attention, processing speed, and executive functioning, domains that are essential for effective communication [38]. Reduced attentional capacity may impair the processing of incoming linguistic information, whereas deficits in executive control may contribute to disorganized speech, reduced verbal fluency, and difficulties maintaining coherent discourse [39].

### 4.2. Mediterranean Dietary Pattern and Neuroprotective Effects

In contrast to Western dietary patterns, the Mediterranean diet has been associated with more favorable neurocognitive and behavioral outcomes in children and adolescents [40]. This dietary pattern is characterized by high consumption of fruits, vegetables, whole grains, legumes, dry nuts, olive oil, and fish, providing a rich source of antioxidants, polyphenols, dietary fibers, and omega-3 acids [41].

Adherence to the Mediterranean diet has been associated with lower prevalence of ADHD symptoms and improved cognitive performance [42]. The neuroprotective effects of this dietary pattern are thought to be mediated through its anti-inflammatory and antioxidant properties, as well as its capacity to support gut microbiome diversity and metabolic homeostasis [43].

These mechanisms may also exert downstream effects on communication-related functions. Improved functioning enhanced attentional control, and better emotional regulation may facilitate more efficient language processing, narrative organization, and social communication [38]. Thus, adherence to the Mediterranean diet may contribute not only to overall cognitive health but also to domains directly relevant to communication performance in individuals with ADHD [44].

Although additional nutritional strategies, such as elimination diets and targeted supplementation protocols have been explored in ADHD, available findings remain inconsistent and fall outside the main focus of the present review [45].

### 4.3. Dietary Habits, Behavioral Regulation, and Cognitive Stability

Beyond overall dietary patterns, specific eating behaviors may also influence neurocognitive functioning in ADHD [46]. Disrupted eating routines, habitual snacking, and excessive consumption of foods rich in sugar may interfere with metabolic stability and neurochemical balance. These alterations may exacerbate attentional instability and emotional dysregulation, both of which represent core features in ADHD [47].

Rapid fluctuations in blood glucose may affect sustained attention, impulsivity, and emotional regulation. Any associated changes in conversational behaviour, such as difficulties with turn-taking or topic maintenance, would most likely occur through these broader cognitive and behavioral effects rather than through a specific language or communication mechanism [14,47,48].

These observations highlight the importance of considering not only what individuals eat, but also how and when they eat, in shaping cognitive and behavioral outcomes relevant to communication.

### 4.4. Dietary Patterns as Upstream Modulators of Communication Outcomes

Taken together, current evidence suggests that dietary patterns may act as upstream modulators influencing neurocognitive processes fundamental to communication. Diet-related influences on attention, executive functioning, and emotional regulation may indirectly affect cognitive and communicative processes involved in language and social interaction [49].

Although direct evidence linking specific dietary patterns to communication difficulties in ADHD remains limited, existing findings supports a conceptual framework in which nutrition may influence communication through its effect on brain function and neurocognitive regulation. This perspective highlights the importance of considering dietary factors within a broader biopsychosocial framework of communication impairments in ADHD [50].

## 5. Micronutrients and Neurobiological Substrates of Communication

### 5.1. Iron, Zinc, and Magnesium: Roles in Neurotransmission and Cognitive Function

Essential trace elements, including iron, zinc, and magnesium, play important roles in neurodevelopment and neurotransmitter regulation, processes that have been implicated in the pathophysiology of ADHD [51]. Iron is particularly important for dopamine synthesis and regulation, serving as a cofactor for tyrosine hydroxylase, the rate-limiting enzyme in catecholamine production [52]. Reduced ferritin concentration has been reported in individuals with ADHD and have been associated with greater symptom severity [53].

Zinc contributes to synaptic plasticity and modulates both glutamatergic and dopaminergic signaling, whereas magnesium is involved in neuronal excitability and regulation of *N*-methyl-D-aspartate (NMDA) receptor activity [54,55]. As a consequence, deficiencies in these micronutrients may adversely affect signaling and cognitive functioning [56].

From a communication perspective, disruption in dopaminergic and glutamatergic neurotransmission may influence cognitive and linguistic processes involved in speech production, verbal fluency, and auditory processing [57]. Impaired neurotransmitter function may also contribute to difficulties sustaining attention during language-related tasks, potentially affecting both expressive and receptive communication abilities [58].

### 5.2. B-Complex Vitamins and Vitamin D: Neurodevelopmental and Neural Connectivity

Vitamins of the B complex, including B6, B12, and folate, are essential for one-carbon metabolism, myelination, and neurotransmitter synthesis. These processes are fundamental for efficient neural communication and brain connectivity [59]. Deficiencies in B vitamins have been associated with impaired cognitive function, reduced processing speed, and alterations in mood and behavior [60].

Vitamin D has also emerged as an important neuroactive compound, involved in neurodevelopment, immune modulation, and regulation of neurotrophic factors [61]. Decreased levels of vitamin D have been reported in children with ADHD and may be associated with increased symptom severity [62].

These micronutrients may influence communication-related brain function by supporting neural integrity and signal transmission [59]. Disruption in myelination and synaptic efficiency may impair the speed and accuracy of language processing, affecting comprehension, verbal output, and higher-level discourse organization [63].

### 5.3. Antioxidant Functions of Micronutrients and Neuroprotection

Beyond their role in neurotransmission and neurodevelopment, several micronutrients contribute to antioxidant defense mechanisms. Elements such as zinc, selenium, and certain vitamins act as cofactors for antioxidant enzymes, helping to neutralize reactive oxygen species (ROS) and maintain redox balance [64].

In the context of ADHD, increased oxidative stress and reduced antioxidant defenses have been consistently reported, suggesting that micronutrient deficiencies may further increase neuronal vulnerability [65]. Oxidative damage may impair synaptic function, mitochondrial activity, and neuroplasticity, alla of which are essential for cognitive and language-related processes [66].

Given the high metabolic demands of neural networks involved in language processing, impaired antioxidant defenses may indirectly contribute to communication difficulties, particularly in tasks requiring rapid processing and integration of auditory and linguistic information [67].

In addition to the micronutrients discussed above, trace elements such as selenium, and copper may contribute to neurobiological processes relevant to ADHD [68]. Selenium is an essential component of antioxidant defense systems, particularly through its role in glutathione peroxidase activity, and may help protect against oxidative stress-induced neural damage [69]. Copper, in contrast, participates in several enzymatic reaction involved in neurotransmitter metabolism, including the conversion of dopamine to norepinephrine by dopamine β-hydroxylase [70].

Notably, the balance between zinc and copper levels has been suggested to influence outcomes, with alterations in the zinc/copper ratio potentially contributing to ADHD symptomatology [71]. Although direct evidence these trace elements to communication outcomes is currently lacking, their involvement in oxidative balance, neurotransmitter regulation, and neuronal signaling has been well documented [72]. Consequently, these mechanisms may indirectly influence cognitive and language-related processes relevant to communication.

The micronutrients reviewed in this section influence neurodevelopment and communication-related processes through multiple interconnected biological pathways. As summarized in Table 1, these mechanisms can be broadly categorized into neurotransmitter regulation, neurodevelopment and neural integrity, and oxidative stress modulation.

### 5.4. Clinical Evidence and Limitation of Micronutrient Supplementation

Variable findings have been found after clinical studies examining micronutrient supplementation in ADHD. Although some randomized controlled trials and metanalyses have reported modest improvements in behavioral symptoms regarding the use of iron, zinc, or multinutrient formulations, the generalizability and consistency of their effects differs in a big margin across studies [73,74].

These inconsistencies may be attributable to heterogeneity in study design, baseline nutritional status, intervention dosage, treatment duration, and outcome measures. It should be noted that the majority of studies have focused on core ADHD symptoms, while communication-related outcomes have received comparatively little attention [3].

Despite increasing interest in micronutrient supplementation in ADHD, communication-related outcomes have rarely been examined. Although existing biological evidence supports a potential link between micronutrients and communication-related functions, empirical studies directly evaluating this relationship remain limited [74].

### 5.5. Micronutrients as Modulators of Communication-Relevant Neural Processes

Collectively, available evidence suggests that micronutrients act as modulators of neurobiological processes that support communication. By influencing neurotransmission, neural network function, and oxidative homeostasis, micronutrient status may indirectly affect language development, speech processing, and pragmatic aspects of communication [75].

Within this framework, communication difficulties in ADHD may reflect not only cognitive dysfunction but also underlying biological alteration influenced by nutritional status. As a consequence, nutritional status may represent an important component of the multifactorial mechanisms contributing to communication-related difficulties in ADHD [76].

## 6. Polyunsaturated Fatty Acids (PUFAs)

### 6.1. Role of Polyunsaturated Fatty Acids (PUFAs) in Brain Structure and Function

Polyunsaturated Fatty Acids (PUFAs), particularly the omega-3 fatty acids docosahexaenoic acid (DHA) and eicosapentaenoic acid (EPA), play important roles in brain development and neural function [77]. DHA is a major structural component of neuronal membranes and contributes to membrane fluidity, synaptic plasticity, and intracellular signaling [78]. EPA, although present in lower concentration within the central nervous system (CNS), is involved in the regulation of inflammatory pathways and neuronal signaling processes [79].

Adequate availability of omega-3 fatty acids is important for several neurodevelopmental processes, including development of neuronal networks, synaptogenesis, and myelination [80]. Conversely, an imbalance between omega-6 and omega-3 fatty acids, a pattern frequently associated with Western dietary habits, has been linked to alteration in neural function and an increased vulnerability to neurodevelopmental disorders [81].

### 6.2. Evidence of PUFA Alteration in ADHD

Evidence from observational studies indicates that individuals with ADHD often exhibit lower circulating concentrations of omega-3 fatty acids compared with typically developing peers [82]. Findings from intervention studies further suggest that omega-3 supplementation may provide modest benefits for ADHD symptom management, with the most consistent effects reported for attentional functioning [83].

Interpretation of the available evidence is complicated by substantial variation across studies, including differences in participants characteristics, supplementation protocols, treatment duration, and baseline nutritional status [84]. Although omega-3 fatty acids are not considered a stand-alone treatment for ADHD, they may represent a useful complementary approach within a broader management strategy [85].

### 6.3. Mechanisms Linking PUFAs to Neurocognitive Function

Multiple mechanisms have been proposed to explain the potential effects of PUFAs in ADHD. Omega-3 fatty acids contribute to the regulation of neuroinflammatory responses by limiting the production of pro-inflammatory mediators and promoting the resolution of inflammation [86]. In addition, they influence neuronal communication through their effects on dopaminergic and serotonergic neurotransmission, systems that play central roles in attentional control, executive functioning, and behavioral regulation [87].

Beyond their effects on neuroinflammation and neurotransmitter regulation, PUFAs contribute to the maintenance of synaptic plasticity and the integrity of neuronal networks, both of which are essential for efficient neural communication [88]. By supporting these neurobiological processes, omega-3 fatty acids may promote cognitive functions that are frequently impaired in ADHD, including attentional control, working memory, and executive functioning [84].

### 6.4. Relevance of PUFAs to Communication-Related Processes

In addition to their role to cognitive functioning, PUFAs may influence neural systems that support language and communication [89]. DHA is enriched in cortical regions involved in auditory and language processing, where it contributes to neuronal membrane function and synaptic signaling [90]. Adequate availability of omega-3 fatty acids may therefore facilitate the maturation and functional integrity of neural networks responsible for speech perception, auditory discrimination, and language development [91].

Insufficient availability of omega-3 fatty acids may compromise the efficiency of neural networks responsible for auditory and linguistic processing, potentially affecting both language comprehension and verbal expression [89]. Moreover, because omega-3 fatty acids influence attentional regulation, working memory, and executive functioning, they may also have indirect effects on communication. Improvements in these cognitive domains could facilitate the planning and organization of verbal responses, support coherent discourse, and enhance the ability to adjust communication according to social and contextual demands [39].

Overall, the availability evidence indicates that PUFAs may influence communication-relevant processes in ADHD through their effects on neuroinflammation, neurotransmission, and higher-order cognitive functions [8]. Although the clinical benefits observed with omega-3 supplementation have generally been modest, their favorable safety profile and mechanistic rationale support their potential role within a comprehensive management strategy for ADHD [92]. These observations reinforce the concept that nutritional factors can modulate biological pathways involved in communication and language-related functioning. Nevertheless, evidence specifically addressing communication outcomes remains limited, as most intervention studies have prioritized behavioral symptoms over standardized assessments of language, speech, or social communication.

## 7. Gut Microbiome and the Gut–Brain–Communication Axis

### 7.1. Alterations of the Gut Microbiome in ADHD

The gut microbiome has emerged as a key regulator of neurodevelopment and brain function through its involvement in the gut–brain axis [93]. Growing evidence suggests that individuals with ADHD exhibit alteration in gut microbial composition, commonly referred to as dysbiosis [94].

Several studies have reported reduced microbial diversity and alteration in the relative abundance of specific bacterial taxa in individuals with ADHD, although findings remain heterogenous across populations and study designs [95,96]. These variations may reflect differences in dietary habits, medication use, environmental exposures, age, and other host-related factors [96]. Nevertheless, the consistent observation of gut microbiome alterations supports the hypothesis that dysbiosis may contribute to the pathophysiology of ADHD through microbiota–gut–brain axis (MGBA) interactions [97].

### 7.2. Mechanisms of the Gut–Brain Axis

Communication between the gut microbiome and the CNS is orchestrated through an integrated network of neural, immune, endocrine, and metabolic signalling pathways [98]. A key component of this interaction is the generation of microbial metabolites, particularly short-chain fatty acids (SCFAs), which contribute to the regulation of neuroinflammatory processes, neurotransmitter activity, and blood–brain barriers homeostasis [99]. The intestinal microbiota also influences the synthesis, metabolism, and bioavailability of several compounds, including serotonin, dopamine, and gamma-aminobutyric acid (GABA), thereby affecting neural functions involved in attention, cognition, and behavioral regulation [31,100]. In addition, vagal afferent signaling provides a direct communication route through which gut-derived signals can modulate CNS activity [101].

Disruption of intestinal barrier function may permit the passage of microbial components and inflammatory mediators into the systemic circulation, promoting immune activation and neuroinflammatory responses that have been implicated in ADHD pathophysiology [102]. Because these biological processes affect neural systems involved in attentional regulation, executive functioning and auditory-linguistic processing, gut microbial dysbiosis may also have downstream effects on communication-related functions [103]. Although direct evidence linking microbiome alterations to communication impairments in ADHD is still limited, the biological mechanisms described above provide a plausible explanation for how gut dysbiosis may influence communication-related functions.

### 7.3. Microbiome, Neurodevelopment and Cognitive Function

The biological interactions occurring along the gut–brain axis extend beyond immune regulation and influence fundamental aspects of brain maturation and cognitive function [104]. Alteration in the composition and metabolic activity of the gut microbiota may affect synaptic remodeling, neuronal network organization, and neurochemical homeostasis, thereby influencing neural processes that support cognition [105].

In individuals with ADHD, disturbances in these microbiome-related pathways may contribute to impairments in attentional control, executive functioning, and emotional regulation [106]. Because these cognitive functions provide the foundation for effective language use and social communication, gut microbial dysbiosis may also have indirect consequences for communication-related abilities, particularly in situations requiring complex linguistic processing and adaptive social interaction [107,108,109].

The influence of the gut microbiome appears to be particularly important during early neurodevelopment, when microbial colonization and environmental exposures interact to shape brain maturation [110]. Dietary habits, especially during critical development periods, may therefore contribute to long-term neurodevelopmental outcomes by modifying the composition and functional activity of the intestinal microbiota [111].

Key microbiome-related mechanisms and their potential relevance to communication in ADHD are summarized in Table 2.

### 7.4. The Gut Microbiome and Communication-Related Processes

Beyond contribution to cognitive functioning, the gut microbiome may also influence neurobiological processes that support communication [112]. Experimental and clinical evidence indicates that microbiome-related mechanisms can affect social behavior, emotional regulation, and sensitivity to social cues, all of which are essential components of pragmatic communication [113]. Given the prominent role of these functions in successful interpersonal interactions, alterations in gut microbial composition may indirectly contribute to communication difficulties frequently observed in individuals with ADHD.

Microbiome-associated alterations in serotonergic and dopaminergic signaling may extend beyond behavioral regulation to influence neural processes involved in language and communication [114]. Likewise, immune dysregulation and neuroinflammatory responses associated with gut microbial imbalance may interfere with the function of brain networks responsible for auditory processing and language comprehension, providing a potential pathway through which gut dysbiosis could contribute to communication difficulties in ADHD [115].

Overall, current evidence supports a biologically plausible link between gut microbial dysbiosis and communication-related functions in ADHD through interconnected neuroimmune, neurochemical and cognitive mechanisms [102]. Although direct clinical evidence remains limited, the pathways discussed above provide a coherent framework for understanding how alterations in gut microbiome may contribute to communication difficulties in individuals with ADHD.

### 7.5. Microbiome-Targeted Intervention and Emerging Evidence

Advances in our understanding of the gut–brain axis have stimulated interest in microbiome-targeted interventions as potential adjunctive approaches for ADHD [116]. Strategies including probiotic and prebiotic supplementation, as well as dietary interventions designed to promote a more diverse and metabolically active gut microbiota, have been investigated for their potential to influence neurobehavioral outcomes [117].

Clinical investigations have suggested that probiotic supplementation may offer benefits for selected behavioral manifestations of ADHD; however, interpretation of these findings is complicated by differences in study design, intervention protocols, and sample characteristics [118]. This growing body of research has also led to the emergence of the concept of “psychobiotics”, referring to microorganisms capable of influencing brain function and mental through interactions with the gut–brain axis [119].

Clinical evidence does not yet allow firm conclusions regarding the effects of microbiome-targeted intervention on communication in ADHD [120]. Nevertheless, the biological rational supporting these approaches highlights the gut microbiome as a promising therapeutic target deserving of further clinical investigation.

### 7.6. Microbiome as a Central Link in the Diet–Brain–Communication Axis

The evidence presented throughout this chapter supports the concept that the gut microbiome represents a key biological interface linking dietary factors with communication-related brain function in ADHD [94]. By integrating signals from nutrition, immune regulation, neurotransmitter activity, oxidative homeostasis, and gut–brain communication, the intestinal microbiota may influence multiple neurobiological processes underlying attention, executive functioning, language, and social communication [105]. Rather than acting through a single mechanism, these interconnected pathways provide a framework for understanding how dietary factors may indirectly contribute to communication difficulties in ADHD.

## 8. Oxidative Stress and Neuroinflammation

### 8.1. Oxidative Stress in ADHD

Oxidative stress arises when the generation of reactive oxygen species (ROS) exceeds the capacity of endogenous antioxidant systems to maintain redox homeostasis [121]. A growing body of evidence indicates that oxidative imbalance is more pronounced in individuals with ADHD, as reflected by increased biomarkers of lipid peroxidation and reduced antioxidant defenses [122].

Multiple factors may contribute to this altered redox state, including suboptimal dietary patterns, inadequate intake of antioxidant micronutrients, and metabolic disturbances [123]. Because the developing brain is characterized by high oxygen consumption and substantial metabolic activity, persistent oxidative stress may compromise neuronal integrity and increase susceptibility to cellular dysfunction [124].

### 8.2. Neuroinflammation and Immune Dysregulation

Neuroinflammation has emerged as another important biological process contributing to the neurobiology of ADHD and is closely interconnected with oxidative stress [125]. Altered immune activity, characterized by increased circulating concentrations of pro-inflammatory cytokines, including inteleukin-6 (IL-6) and tumor necrosis factor-alpha (TNF-a) has been described in individuals with ADHD [126].

Multiple mechanisms may contribute to persistent neuroinflammatory activity, including peripheral immune dysregulation, gut microbiome alterations, and impaired intestinal barrier function. These processes can modify neurotransmitter signaling interfere with synaptic plasticity and disrupt the organization of neuronal networks involved in cognitive function [127].

Rather than acting independently, oxidative stress and neuroinflammation reinforce one another through reciprocal biological interactions, creating a self-sustaining cycle that may amplify neuronal dysfunction and contribute to the persistence of ADHD-related neurobiological abnormalities [10].

### 8.3. Effects on Brain Function and Neural Connectivity

Neuroinflammation can cause problems related to neuronal function that extend beyond molecular alterations, thereby affecting the organization and efficiency of large-scale brain networks [128]. Dysfunction of the mitochondria, impaired cellular energy metabolism, and altered synaptic signaling collectively compromise the capacity of neurons to sustain normal cognitive processes [129].

These biological disturbances are likely to affect brain regions involved in higher-order cognitive and communicative functions, including the prefrontal cortex, which supports executive functioning and behavioral regulation, as well as temporal cortical areas responsible for auditory and language processing. Subcortical and brainstem structures may also be involved, potentially influencing sensory integration and speech-related functions [67].

Altered functional connectivity among these distributed neural systems may reduce the efficient integration of cognitive, sensory, and linguistic information, providing a justifiable neurobiological basis for communication difficulties observed in individuals with ADHD [130].

### 8.4. Implications for Communication and Language Processing

The neurobiological consequences of oxidative stress and neuroinflammation are likely to affect cognitive systems that support effective communication. Alterations in executive functioning may interfere with the planning, organization, and regulation of verbal output, whereas dysfunction within auditory and temporal processing networks may compromise language comprehension and speech perception [131].

Inflammatory signalling may also influence social cognition and emotional regulation, two processes that are fundamental for successful pragmatic communication [132]. Consequently, disturbances in these neurocognitive domains may contribute to impairments in expressive and receptive language, reduced discourse coherence, and difficulties adapting communication to different social contexts [133].

### 8.5. Interaction with Dietary and Microbiome Factors in the Diet–Brain–Communication Axis

Dietary factors and the gut microbiome are closely interconnected regulators of oxidative balance and immune homeostasis [134]. Nutritional patterns characterized by a high intake of ultra-processed foods and limited antioxidant availability may favor oxidative damage and persistent inflammatory activity, whereas diets rich in bioactive compounds and essential nutrients support endogenous antioxidant defences and immune regulation [135,136]. Alterations in gut microbial composition may further amplify these processes by promoting pro-inflammatory signalling and disrupting immune homeostasis [137].

Oxidative stress and neuroinflammation represent interconnected biological mechanisms through which dietary factors and the gut microbiome alterations may influence brain function in ADHD. By affecting neural systems involved in attentional regulation, executive functioning, and language processing, these pathways provide a biologically plausible explanation for the communication difficulties frequently observed in individuals with ADHD.

## 9. Integrative Neurobiological Mechanisms Underlying Communication Difficulties in ADHD

Diet represents a major environmental factor through which multiple biological pathways relevant to communication may be influenced in ADHD [102]. Rather than acting through a single mechanism, nutritional exposures initiate a cascade of interconnected processes involving neurotransmitter regulation, immune function, oxidative balance, and the gut microbiome [138]. These interconnected biological pathways ultimately converge on neural networks supporting attention, executive functioning, language, and social communication [139].

Τhe gut microbiome represents a key biological intermediary linking dietary exposures with brain function through the gut–brain axis [140]. By regulating microbial metabolite production, intestinal barrier integrity, immune responses, and neurotransmitter signaling, the gut microbiota integrates nutritional influences with CNS function [141]. Consequently, alterations in microbial homeostasis may amplify downstream inflammatory and oxidative processes that ultimately affect neural systems underlying cognitive and communication functions in individuals with ADHD [94].

Chronic low-grade inflammation represents a key converging pathway through which dietary factors and microbiome alterations may influence neurodevelopment. Immune activation and oxidative stress represent tightly interrelated mechanisms through which dietary factors and microbiome alterations may influence neurodevelopment [28]. Persistent inflammatory signaling can promote oxidative imbalance, while oxidative stress further amplifies neuroinflammatory responses, creating a reciprocal cycle capable of disrupting neuronal homeostasis [142]. Together, these biological processes contribute to alterations in neurotransmission, synaptic plasticity, and mitochondrial function that are relevant to communication-related brain networks [143].

Although these biological mechanisms originate through distinct pathways, they ultimately converge on distributed neural networks involved in communication. Functional alterations affecting prefrontal, temporal, subcortical, and auditory networks may impair executive language control, speech perception, language comprehension, and pragmatic communication. This intersection provides a biologically credible explanation for the communication difficulties frequently observed in individuals with ADHD [3,144].

**Figure 2:**
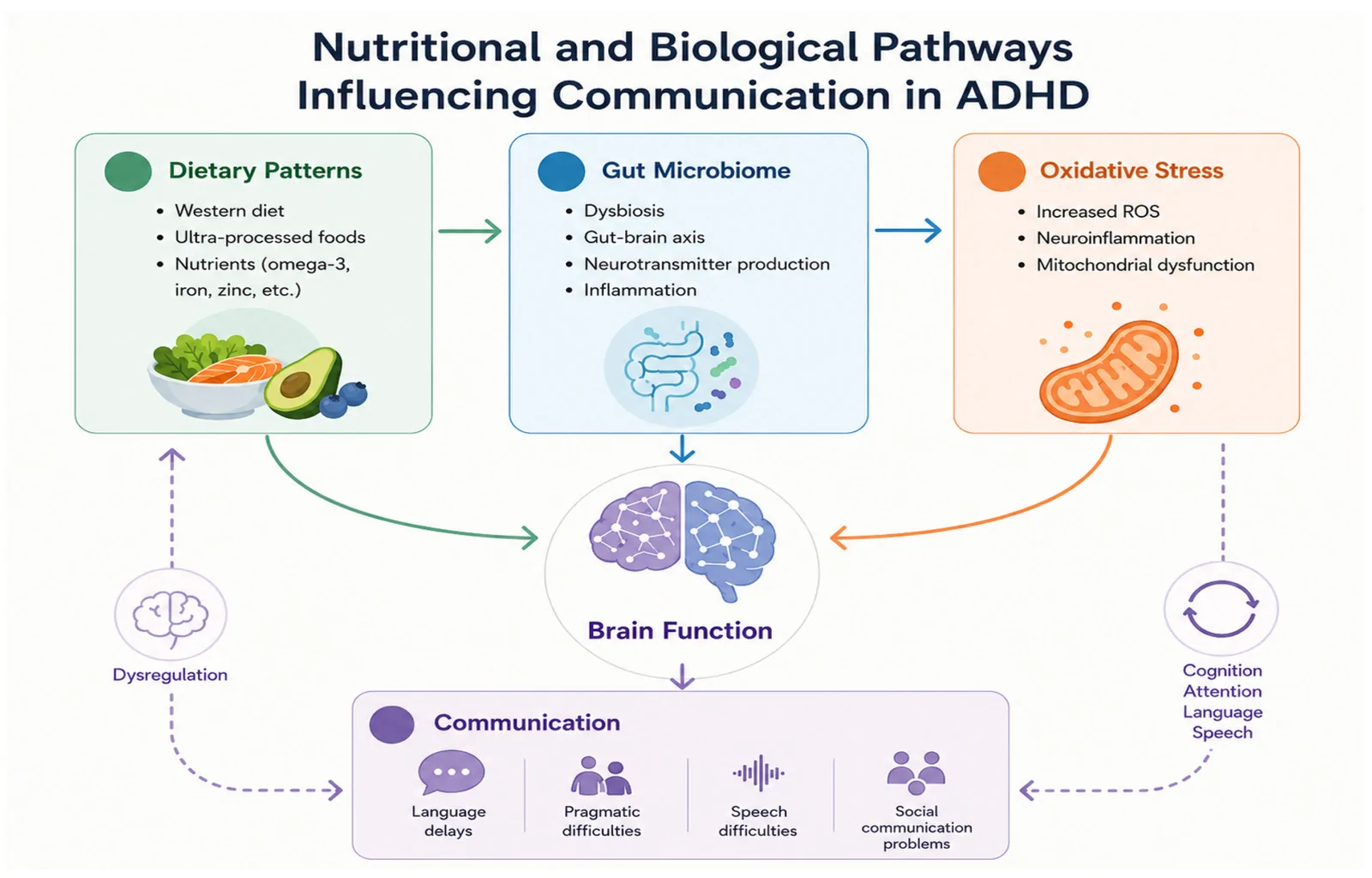
Proposed integrative neurobiological framework linking nutrition to communication-related outcomes in ADHD. Dietary factors serve as upstream environmental modulators influencing the gut microbiome, immune responses, oxidative balance, and neurotransmitter regulation. These interconnected biological processes converge on neural systems supporting attention, executive functioning, language, and social communication, ultimately contributing to communication difficulties in individuals with ADHD. Feedback arrows indicate possible reciprocal relationships within the proposed framework and should not be interpreted as established direct causal pathways.

Altogether, these findings support a model in which nutrition acts as an upstream environmental influence initiating a cascade of interrelated biological events. By modulating the gut microbiome, immune responses, oxidative homeostasis, and neurotransmitter regulation, dietary factors may indirectly shape neural systems supporting communication [141]. Rather than functioning independently, these biological processes operate as an interconnected network that shapes neurodevelopment and communication-related functions in ADHD. The proposed interactions among these biological processes are summarized in Figure 2.

In order to be able to clinically manage ADHD, we have to understand these closely linked mechanisms that offer us important implications. Conventional therapeutic approaches, particularly in individuals presenting communication difficulties, may be complemented by nutritional strategies that are aimed at improving dietary quality and targeting biological pathways such as gut microbiome composition, inflammation, and oxidative balance [145]. While current evidence remains heterogeneous, this integrative framework supports the potential value of individualized nutritional interventions and emphasizes communication outcomes as clinically meaningful targets for future intervention studies [146].

## 10. Nutritional Interventions Targeting Communication Outcomes in ADHD

### 10.1. Elimination Diets and Restrictive Dietary Approaches

Dietary interventions have gathered a lot of attention for how they can be utilized to reduce ADHD symptoms. These include elimination and restrictive diets. Among these, the Few-Foods-Diet (FFD) is one of the most studied approaches. This approach involves temporary restriction of commonly consumed foods that may trigger behavioral or inflammatory responses. Then it is followed by systematic food reintroduction to identify potential dietary sensitivities [147].

Some studies have reported improvements in behavioral symptoms in subsets of children with ADHD following restrictive dietary interventions [148]. However, findings remain heterogeneous, and methodological limitation, including small sample sizes, variability in dietary protocols, and lack of long-term follow-up, limit the generalizability of results [146].

From a communication perspective, improvements in attentional regulation and behavioral control following dietary modification may indirectly facilitate language processing, conversational organization, and social communication. Despite these promising findings, direct evidence specifically examining communication outcomes remains limited [5].

### 10.2. Micronutrient and Omega-3 Supplementation

Supplementation with micronutrients has been investigated as a supportive intervention in ADHD management [149]. Iron, zinc, magnesium, vitamin D, and multinutrient formulations have shown beneficial effects in selected populations, particularly in individuals with documented deficiencies [150].

Similarly, omega-3 fatty acid supplementation, especially formulations containing DHA and EPA, has been associated with small improvements in attention and behavioral regulation [151]. The proposed mechanisms include modulation of neurotransmitter systems, reduction in neuroinflammation, and enhancement of synaptic plasticity [152].

These neurobiological effects may also have indirect implications for communication abilities. By improving attention, working memory, and executive functioning, these interventions may indirectly support speech organization, receptive language processing, and pragmatic communication skills. However, communication-specific outcomes have rarely been assessed as primary endpoints in intervention studies.

### 10.3. Microbiome-Targeted Interventions

Increasing understanding of the gut–brain axis has led to growing interest in microbiome-targeted interventions, including probiotics, prebiotics, and psychobiotics. These approaches aim to modulate gut microbial composition and reduce inflammatory and oxidative processes associated with neurodevelopmental dysfunction [112].

Preliminary studies suggest that probiotics may provide potential benefits for emotional regulation, behavioral symptoms, and cognitive function [141]. Proposed mechanisms include modulation of immune signaling, neurotransmitter production, and intestinal barrier integrity [153].

Although evidence remains preliminary, microbiome-targeted interventions may also influence communication-related domains, particularly pragmatic communication and social interaction, through their effects on emotional regulation and social cognition [154]. However, communication-specific outcomes remain largely unexplored in intervention studies, limiting conclusions regarding their clinical utility.

Overall, nutritional interventions should be regarded as adjunctive rather than standalone therapeutic strategies in ADHD. While current evidence remains heterogeneous, available findings suggest that dietary approaches may contribute to broader multidisciplinary management, particularly when communication outcomes are considered alongside behavioral and cognitive outcomes.

## 11. Pharmacological Treatment, Appetite, and Communication Development

### 11.1. Pharmacological Management in ADHD

Pharmacological treatment remains one of the primary therapeutic approaches for ADHD, with stimulant medications such as methylphenidate and amphetamine derivatives being widely used. These agents primarily target dopaminergic and noradrenergic pathways, contributing to improvements in attention, impulse control, and behavioral regulation [155].

Non-stimulant medications, including atomoxetine and a2-adrenergic agonists, are also used in selected cases, particularly when stimulants are ineffective or poorly tolerated. Although pharmacological intervention is generally effective in reducing core ADHD symptoms, their broader effects on communication-related outcomes remain less clearly understood [156].

### 11.2. Appetite Suppression and Nutritional Implications

One of the most frequently reported adverse effects of stimulant medications is appetite suppression, which may lead to reduced caloric intake and altered nutritional status. Long-term treatment has also been associated with concerns regarding growth velocity and weight gain in some children [157].

Reduced appetite may contribute to inadequate intake of essential nutrients, including iron, zinc, omega-3 fatty acids, and vitamins involved in neurodevelopmental processes [49]. Considering the proposed role of these nutrients in neurotransmission, neural connectivity, and oxidative balance, medication-related nutritional changes may have indirect implications for cognitive and communication functions [59].

Children with ADHD may already present with selective eating behaviors or sensory-related food aversions, potentially increasing vulnerability to nutritional imbalance during pharmacological treatment [158].

### 11.3. Pharmacological Effects on Communication and Cognitive Function

Improvements in attentional regulation following pharmacological treatment may positively influence communication abilities by enhancing listening capacity, conversational engagement, and organization of speech. Improved executive control may also support narrative coherence and pragmatic communication [144].

Although communication outcomes have rarely been evaluated directly in pharmacological studies, limited evidence suggests that stimulant treatment may improve certain functional aspects of communication, such as narrative organization, primarily through enhanced attention and executive functioning [159].

Furthermore, medication-related nutritional changes may interact with biological pathways implicated in communication and neurodevelopment, including oxidative stress and neurotransmitter regulation [160].

Overall, pharmacological treatment and nutritional status should be considered complementary rather than independent components of ADHD management. Monitoring appetite, growth, and nutritional status may help optimize long-term functional outcomes, particularly when communication and broader developmental needs are considered.

## 12. Clinical Implications for Communication Assessment and Intervention in ADHD

### 12.1. Communication Difficulties in ADHD: A Multidimensional Perspective

Communication difficulties in ADHD should not be interpreted solely as secondary consequences of inattention or behavioral dysregulation [161]. Rather, current evidence suggests that communication impairments may arise through the complex interaction of cognitive, neurobiological, nutritional, and environmental factors, contributing to considerable heterogeneity in clinical presentation [76].

Recognizing this complexity has important implications for clinical assessment. Communication difficulties may extend beyond structural language deficits to include impairments in executive language functions, pragmatic communication, auditory processing, emotional regulation, and social cognition [133]. Consequently, comprehensive evaluation should consider the multiple biological and functional dimensions that may influence communication performance in individuals with ADHD [161].

### 12.2. Importance of Multidisciplinary Assessment

Given the multidimensional nature of communication difficulties in ADHD, comprehensive assessment should extend beyond the evaluation of language abilities alone. Collaboration among developmental pediatricians, speech-language therapists, psychologists, dietitians, and other healthcare professionals may facilitate a more comprehensive characterization of each individual’s communication profile and associated biological and behavioral factors [162,163].

Nutritional assessment may be particularly relevant in individuals presenting with selective eating behaviors, restrictive dietary patterns, gastrointestinal symptoms, or suspected micronutrient deficiencies. Consideration of dietary habits and lifestyle factors may provide additional clinical insights into potentially modifiable mechanisms contributing to communication difficulties and support the development of individualized management strategies [164].

### 12.3. Implications for Speech-Language Therapy

Speech-language therapy in ADHD should address not only structural language abilities but also higher-order communication skills, including pragmatic language, discourse organization, conversational competence, and social communication skills. These domains are frequently influenced by attentional regulation, executive functioning, and social-cognitive processes, requiring intervention approaches that extend beyond traditional language-based techniques [165].

Although current evidence does not support nutritional interventions as substitutes for speech-language therapy [76], the integrative framework presented in this review suggests that nutritional status may represent an important contextual factor influencing therapeutic engagement and overall communication performance. Consequently, consideration of nutritional health may complement individualized speech-language intervention without replacing evidence-based communication-focused therapies.

From a clinical perspective, the present framework may encourage broader multidisciplinary awareness of factors that could influence general health and functioning in individuals with ADHD. However, nutritional assessment or intervention should be based on an independent clinical indication, such as restrictive eating, gastrointestinal symptoms, growth concerns, or suspected nutrient deficiency. Communication assessment and evidence-based speech-language intervention should remain the primary approaches when communication difficulties are present.

## 13. Quality Assessment

Two independent external assessors, neither of whom was a member of the author team or involved in preparing the manuscript, evaluated the review using the SANRA scale. Following discussion and agreement on the individual domains, the final consensus score was 12/12. The detailed assessment is presented in Supplementary Table S1.

The manuscript clearly defines its objectives, addresses a clinically relevant topic, and it is supported by a transparent literature search strategy as well as a balanced synthesis of current evidence. The structure of the manuscript, together with the inclusion of summary tables and integrative conceptual figures, further strengthens the clarity and presentation of the review. The detailed SANRA evaluation is provided in Supplementary Table S1.

## 14. Limitations

Despite the growing body of evidence linking nutrition, gut microbiome alterations, oxidative stress, and neurodevelopmental outcomes in ADHD, several important limitations should be acknowledged. First, much of the available evidence derives from observational studies, limiting the ability to establish causal relationships among nutritional factors, biological pathways, and ADHD-related outcomes.

A major limitation of the current literature is the relative scarcity of communication-specific outcome measures. Most studies investigating nutritional or microbiome-related factors in ADHD primarily focus on behavioral symptoms, attention or executive functioning, whereas expressive language, receptive language, pragmatic communication, and speech processing are rarely evaluated. Consequently, many of the proposed associations between biological pathways and communication difficulties remain indirect and are largely based in theoretical integration rather than direct empirical evidence. Therefore, findings concerning core ADHD symptoms, attention, executive functioning, or biological markers should not be generalized directly to communication outcomes. In the present review, these findings are used mainly to support the biological plausibility of the proposed framework.

Furthermore, substantial methodological heterogeneity exists across studies with respect to participant characteristics, dietary assessment methods, microbiome analysis techniques, supplementation protocols, and outcome measures. This variability complicates comparisons across studies and limits the generalizability of current findings.

An additional challenge is the complexity of the biological pathway discussed throughout this review. Nutrition, gut microbiome, immune activation, oxidative stress, neurotransmitter regulation, and neurodevelopment are highly interconnected processed that are unlikely to operate independently. As a result, the relative contribution of each pathway to communication impairments in ADHD remains difficult to determine, and their interactions require further clarification.

As a narrative review, this work is also subject to the inherent limitation of narrative synthesis, including potential selection bias and the absence of quantitative meta-analytic evaluation. Nevertheless, methodological rigor and reporting transparency were enhanced through adherence to the Scale for the Assessment of Narrative Review Articles (SANRA). Accordingly, the conclusion presented in this review should be interpreted as an integrative synthesis of the current evidence rather than definitive evidence of causal relationships.

## 15. Future Directions

Future research should prioritize longitudinal and well-designed interventional studies to clarify the complex relationships among nutrition, gut microbiome composition, oxidative stress, and communication outcomes in ADHD. Establishing causal pathways will be essential for determining whether modulation of these biological processes can meaningfully influence communication development.

A major priority should be the incorporation of standardized communication outcome measures into future nutritional and microbiome research. Expressive and receptive language, pragmatic communication, discourse organization, and speech processing should be evaluated alongside behavioral, cognitive, and neuropsychological outcomes to better characterize the relationship between biological mechanisms and communication functioning.

Future investigations should also focus on identifying biological and clinical subgroups that may differ in their responsiveness to nutritional and microbiome-targeted interventions. Advances in multi-omics technologies, biomarker discovery, and microbiome profiling may facilitate more precise characterization of individual neurodevelopmental profiles and contribute to the development of personalized intervention strategies.

Finally, future intervention studies should evaluate multidisciplinary models integrating nutritional optimization, microbiome-targeted therapies, pharmacological management, and speech-language intervention. Such approaches may provide a more comprehensive understanding of how biological, cognitive, and environmental factors can interact to influence communication development in ADHD.

## 16. Comparison with Previous Reviews and Contribution of the Present Review

Several previous reviews overlap with parts of the present topic, but they differ in their scope, methods, and primary outcomes. Pinto et al. [45] mainly examined dietary patterns and dietary interventions in ADHD. Lewis et al. [7] focused on the relationships among diet, gut dysbiosis, inflammation, and oxidative stress. Visternicu et al. [166] examined oxidative stress, antioxidant systems, the gut–brain axis, and diet in relation to ADHD symptoms and comorbid conditions. Ghosh and Singh [167] provided a more focused systematic review and meta-analysis of gut microbiome differences in children with ADHD and the possible value of dietary and probiotic interventions. The main characteristics and conclusions of these reviews are compared with those of the present review in Table 3.

The findings of the present review are broadly consistent with earlier reviews in showing that dietary patterns, gut microbiome alterations, oxidative stress, and inflammation may form part of the wider biological context of ADHD. The reviews also agree that nutritional and microbiome-targeted approaches should be viewed as supportive strategies rather than replacements for established ADHD treatments. Pinto et al. reported that unhealthy dietary patterns were associated with ADHD and concluded that stronger evidence was needed before dietary interventions could be routinely implemented. Lewis et al. similarly emphasized the possible interaction between diet, dysbiosis, inflammation, and oxidative stress, while noting the limited direct evidence for dietary interventions in ADHD. Visternicu et al. further supported the relevance of oxidative stress, antioxidant defenses, and the gut–brain axis. Ghosh and Singh added quantitative evidence of gut microbiome differences in children with ADHD and identified probiotics and fiber-rich diets as possible adjunctive approaches. These conclusions are therefore consistent with the biological pathways included in the present framework.

The main difference lies in the outcome being examined. Previous reviews primarily addressed ADHD symptoms, dietary risk, biological markers, microbiome composition, and treatment response. In contrast, the present review places communication difficulties at the center of the synthesis. It connects expressive and receptive language, pragmatic communication, speech and auditory processing, discourse organization, and social communication with executive, neural, nutritional, microbial, oxidative, and inflammatory mechanisms. None of the four overlapping reviews examined communication outcomes as their primary focus.

The present review therefore extends rather than duplicates the earlier literature by identifying communication as an underexamined clinical outcome and by proposing directions for communication-focused assessment and future intervention studies. However, because direct studies linking these biological factors with communication outcomes in ADHD remain scarce, the proposed framework should be interpreted as an evidence-informed conceptual synthesis rather than as evidence of causal relationships.

## 17. Conclusions

Communication difficulties in ADHD should no longer be viewed solely as secondary consequences of inattention or executive dysfunction. Rather, the available evidence supports a multifactorial model in which cognitive, neurobiological, nutritional, microbiome-related, and environmental factors interact to influence communication development and functioning.

This narrative review integrates current evidence linking dietary patterns, micronutrient status, gut microbiome alterations, oxidative stress, immune regulation, and neurotransmitter function with communication-related processes in ADHD. By bringing together these diverse biological pathways within a single conceptual framework, the review highlights communication as a clinically relevant, yet underexplored, outcome in nutritional and neurodevelopmental research.

Although the current evidence remains heterogeneous and largely observational, the findings suggest that nutritional and microbiome-related factors may represent potentially modifiable contributors to communication difficulties in selected individuals with ADHD. This perspective encourages a shift from viewing communication difficulties exclusively as behavioral manifestations toward recognizing their potential biological and environmental determinants. Based on the currently available evidence, nutritional interventions should be regarded as supportive strategies that may complement, but not replace, established evidence-based approaches, including speech-language therapy and standard ADHD management.

Ultimately, this review emphasizes the importance of adopting multidisciplinary and individualized perspectives when addressing communication difficulties in ADHD. Future research integrating communication-specific outcome measures with nutritional, microbiome, and neurobiological investigations will be essential for establishing causal relationships and translating emerging biological insights into evidence-based clinical practice.

## Figures and Tables

**Table 1:** Micronutrient-related biological mechanisms in ADHD and potential relevance to communication.

Micronutrient	Biological Mechanism	Evidence in ADHD	Potential Communication Relevance
Iron	Dopamine synthesis and regulation; myelination	Reduced ferritin levels associated with greater symptom severity	Attention, verbal fluency, language processing, auditory processing
Zinc	Modulation of dopaminergic and glutanergic signaling; synaptic plasticity	Altered zinc status reported in subsets of individuals with ADHD	Executive functioning language; communication efficiency
Magnesium	NMDA receptor regulation and neuronal excitability	Deficiencies reported in some ADHD populations	Sustained attention and language-related information processing
Vitamin 6	Neurotransmitter synthesis	Emerging evidence of involvement in neurobehavioral regulation	Cognitive and language-related functions
Vitamin 12	One-carbon metabolism and myelination	Neurodevelopmental relevance	Neural integrity supporting communication
Folate	DNA synthesis, methylation and neurodevelopment	Implicated in neurodevelopmental processes	Language-related cognitive functions
Vitamin D	Synaptic function and neural signaling	Lower circulating levels frequently reported in ADHD	Communication-relevant neural processes
Selenium	Antioxidant defense and oxidative stress regulation	Limited but emerging evidence	Indirect effects through neuroprotection
Copper	Catecholamine metabolism	Altered Zn/Cu ration associated with ADHD symptomatology	Potential indirect effects through neurotransmitter regulation

Abbreviations: ADHD, attention deficit/hyperactivity disorder; NMDA, *N*-methyl-d-aspartate; Zn/Cu, zinc to copper ratio.

**Table 2:** Key microbiome-related mechanisms and their potential relevance to communication in ADHD.

Microbiome Component/Mechanism	Biological Role	Relevance in ADHD	Potential Impact in Communication
Microbial diversity (dysbiosis)	Maintains ecosystem stability and metabolic balance	Reduced diversity reported in ADHD	May affect global brain function indirectly impacting language and cognitive processing
Short-chain fatty acids (SCFA)	Regulate inflammation, blood–brain barrier, and neuronal signaling	Altered SCFA profiles in ADHD	May influence neural processing speed and integration of linguistic information
Serotonin production (gut-derived)	Modulates mood, cognition, and social behavior	Dysregulated serotonergic signaling in ADHD	May affect social communication and pragmatic language
Dopamine modulation	Influences reward, attention, and executive function	Core neurotransmitter system in ADHD	Affect speech initiation, verbal fluency, and attentional control during communication
Gut permeability (“leaky gut”)	Control passage of metabolites and immune signals	Increased permeability linked to inflammation	May contribute to neuroinflammation affecting language-related brain regions
Immune activation	Mediates inflammatory responses	Elevated inflammatory markers in ADHD	May impair cognitive and linguistic processing
Vagus nerve signaling	Bidirectional gut–brain communication	Altered signaling may affect behavior	Influences emotional regulation and social aspects of communication
Psychobiotics (probiotics/Prebiotics)	Modulate microbiome composition and function	Emerging evidence for behavioral improvement	Potential indirect effects on communication via cognitive and emotional regulation

Abbreviations: ADHD, attention deficit/hyperactivity disorder.

**Table 3:** Comparison of previous overlapping reviews with the present narrative review.

Review	Design and Main Focus	Main Results and Conclusions	Relationship to the Present Review
Pinto et al. [45]	Narrative review of dietary patterns, nutritional supplements, microbiome-targeted interventions, and elimination diets in ADHD	Unhealthy dietary patterns were positively associated with ADHD, whereas healthier dietary patterns were inversely associated. Some benefits were reported for vitamin D in individuals with insufficient or deficient levels and for selected probiotic interventions. Evidence for elimination diets and other dietary interventions remained limited, and caution was recommended.	Supports the relevance of diet and nutritional interventions in ADHD. Communication and language outcomes were not a primary focus.
Lewis et al. [7]	Review of dietary patterns, gut dysbiosis, inflammation, oxidative stress, and the possible therapeutic value of diet in ADHD	Western dietary patterns may contribute to dysbiosis, inflammation, and oxidative stress. The Mediterranean diet and selected nutrients may have supportive value, although direct intervention evidence in ADHD remains limited and heterogeneous.	Closely overlaps with the biological pathways discussed in the present review but focuses mainly on ADHD pathophysiology and symptom management rather than communication outcomes.
Visternicu et al. [166]	Review of oxidative stress, antioxidant defenses, the gut–brain axis, diet, and micronutrients in ADHD	Oxidative imbalance and reduced antioxidant defenses may contribute to ADHD pathophysiology. Diet and the gut–brain axis may influence symptoms and comorbid conditions. Further research on interventions targeting oxidative stress and gut microbiota was recommended.	Supports the inclusion of oxidative stress and the gut–brain axis in the present framework. It does not specifically examine their relationship with communication difficulties.
Ghosh and Singh [167]	Systematic review and meta-analysis of gut microbiome composition and dietary interventions in children with ADHD	The review identified differences in several microbial groups between children with ADHD and neurotypical controls. Probiotics and fiber-rich dietary approaches were proposed as possible supportive strategies for microbial balance and behavioral symptoms.	Provides quantitative support for microbiome alterations in ADHD but has a narrower microbiome-centered scope and does not examine communication-specific outcomes.
Present narrative review	SANRA-guided narrative review integrating dietary patterns, micronutrients, PUFAs, gut microbiome alterations, oxidative stress, neuroinflammation, neural function, and communication difficulties in ADHD	The reviewed pathways may influence communication indirectly through attention, executive functioning, auditory processing, neural connectivity, emotional regulation, and social cognition. Direct communication-specific evidence remains limited.	Extends previous reviews by placing expressive and receptive language, pragmatic communication, speech processing, discourse organization, and social communication at the center of the synthesis.

## Data Availability

No new data were created or analyzed in this study. Data sharing is not applicable to this article.

## Supplementary Materials

The following supporting information can be downloaded at: https://www.mdpi.com/article/10.3390/nu18162664/s1, Table S1. SANRA assessment of the methodological quality of the narrative review. Table S2. Thematic assessment of the consistency and overall level of support for each section of the narrative review. Table S3. Distribution of Evidence by Study Design Across the Major Themes. Table S4. Database-Specific Search Strategies Used in the Narrative Review.

## Abbreviations

The following abbreviations are used in this manuscript:

ADHD	Attention-Deficit/Hyperactivity Disorder
CD	Communication Difficulties
NMDA	*N*-methyl-D-aspartate
ROS	Reactive Oxygen Species
DHA	Docosahexaenoic Acid
EPA	Eicosapentaenoic Acid
CNS	Central Nervous System
MGBA	Microbiota–Gut–Brain Axis
SCFA	short-chain fatty acids
GABA	gamma-aminobutyric acid
TNF-a	Tumor Necrosis Factor-alpha
FFD	Few-Foods-Diet
