# Supplementary material for: Decoding Communication Difficulties in ADHD: A Narrative Review on the Potential Links with Dietary Patterns, the Microbiome, and Oxidative Stress
Source: Nutrients. 2026 Aug 14;18(16):2664. doi: 10.3390/nu18162664 (PMC13516137; doi:10.3390/nu18162664)
Supplement: Supplementary file 1 [file nutrients-18-02664-s001.zip › nutrients-4468431-supplementary.pdf]

Assessment of the Strength of Evidence

Supplementary Table S1. SANRA assessment of the methodological quality of the narrative review.

SANRA domain	Score	Rationale
1. Justification of the article's importance for the readership	2	The review addresses an emerging and clinically relevant topic by integrating evidence on dietary patterns, gut microbiome alterations, oxidative stress, and communication difficulties in ADHD, an area that has not previously been synthesized comprehensively.
2. Statement of concrete aims or formulation of questions	2	The objectives are clearly defined in the Introduction and focus on exploring the biological mechanisms linking nutrition with communication outcomes in ADHD through an integrative perspective.
3. Description of the literature search	2	The review reports the databases searched, eligibility criteria, search concepts, language restrictions, study priorities, and narrative synthesis methodology, providing sufficient methodological transparency.
4. Referencing	2	References are comprehensive, balanced, current, and include landmark publications together with recent systematic reviews, meta-analyses, randomized controlled trials, and high-quality observational studies.
5. Scientific reasoning	2	The review critically evaluates the available evidence, discusses biological plausibility, identifies knowledge gaps, and integrates findings into a coherent mechanistic framework linking nutrition with communication difficulties in ADHD.
6. Appropriate presentation of data	2	Evidence is presented in a logical sequence using thematic organization, summary tables, conceptual models, and clearly structured sections that facilitate interpretation of complex biological mechanisms.
Overall SANRA score	12/12	Excellent methodological quality.

Abbreviations: SANRA, Scale for the Assessment of Narrative Review Articles.

Scoring system: Each SANRA domain is rated from 0 (low standard) to 2 (high standard), resulting in a maximum possible score of 12. The assessment was performed independently by two reviewers, with disagreements resolved through consensus.

Supplementary Table S2. Thematic assessment of the consistency and overall level of support for each section of the narrative review.

Section	Main Topic	Predominant Study Designs	Consistency of Evidence	Overall Level of Support	Research Gaps
3	Communication difficulties in ADHD	Systematic reviews, observational studies, neuroimaging studies	High	High	Few studies directly investigate biological determinants of communication difficulties.
4	Dietary patterns	Meta-analyses, cohort studies, systematic reviews	Moderate–High	Moderate	Limited evidence specifically examining communication outcomes.
5	Micronutrients	RCTs, systematic reviews, meta-analyses	Moderate	Moderate	Considerable heterogeneity regarding supplementation protocols and biomarkers.
6	Polyunsaturated fatty acids (PUFAs)	Meta-analyses, randomized trials	Moderate	Moderate	Small effect sizes and limited communication-specific outcome measures.
7	Gut microbiome	Experimental studies, observational studies, systematic reviews	Moderate	Moderate	Lack of longitudinal and communication-focused studies.
8	Oxidative stress	Case-control studies, biomarker studies, systematic reviews	Moderate	Moderate	Causal relationships remain uncertain.
9	Integrative neurobiological mechanisms	Mechanistic synthesis	Emerging	Emerging	Integrative models require prospective validation.
10	Nutritional interventions	RCTs, systematic reviews	Moderate	Moderate	Communication outcomes rarely included as primary endpoints.
11	Pharmacological treatment and nutrition	Clinical trials, observational studies	Moderate	Moderate	Interaction between medication, nutrition and communication remains poorly investigated.
12	Clinical implications	Expert synthesis based on accumulated evidence	Moderate	Preliminary	Clinical guidelines integrating nutrition and speech-language pathology are lacking.

Abbreviations: ADHD, attention-deficit/hyperactivity disorder; PUFAs, polyunsaturated fatty acids; RCTs, randomized controlled trials.

Supplementary Table S3. Distribution of Evidence by Study Design Across the Major Themes

Section	Systematic Reviews	Meta-analyses	Randomized Controlled Trials	Cohort Studies	Cross-sectional Studies	Mechanistic Studies	Narrative Reviews	Overall Level of support
Communication Difficulties	✓✓✓	✓	✓	✓✓	✓✓	✓✓	✓	High
Dietary Patterns	✓✓	✓✓	✓	✓✓✓	✓✓	✓	✓	Moderate
Micronutrients	✓✓	✓✓	✓✓	✓	✓	✓	✓	Moderate
PUFAs	✓✓	✓✓	✓✓	✓	✓	✓	✓	Moderate
Gut Microbiome	✓✓	✓	✓	✓✓	✓✓	✓✓✓	✓	Moderate
Oxidative Stress	✓✓	✓	✓	✓	✓✓	✓✓✓	✓	Moderate
Nutritional Interventions	✓✓	✓	✓✓	✓	✓	✓	✓	Moderate
Pharmacological Treatment	✓✓	✓	✓✓✓	✓✓	✓	✓	✓	Moderate

Abbreviations: ADHD, attention-deficit/hyperactivity disorder; RCTs, randomized controlled trials.

Note: Checkmarks indicate the relative representation of each study design within the literature considered for the corresponding theme: ✓, limited representation; ✓✓, moderate representation; and ✓✓✓, substantial representation. The symbols do not represent exact study counts or formal methodological-quality ratings. The overall level of support reflects a structured narrative judgment and should not be interpreted as a formal risk-of-bias or certainty-of-evidence assessment. The high rating communication difficulties refers to evidence documenting their presence and characteristics in ADHD, not to direct evidence linking them with nutritional or biological factors.

Supplementary Table S4. Database-Specific Search Strategies Used in the Narrative Review

Database	Search strategy
PubMed	(“attention deficit hyperactivity disorder” OR ADHD) AND (communication OR language OR speech OR pragmatics OR “auditory processing”) AND (diet OR nutrition OR micronutrients OR omega-3 OR microbiome OR “gut-brain axis” OR “oxidative stress” OR inflammation)
Scopus	TITLE-ABS-KEY (“attention deficit hyperactivity disorder” OR ADHD) AND TITLE-ABS-KEY (communication OR language OR speech OR pragmatics OR “auditory processing”) AND TITLE-ABS-KEY (diet OR nutrition OR micronutrients OR omega-3 OR microbiome OR “gut-brain axis” OR “oxidative stress” OR inflammation)
Web of Science	TS=(“attention deficit hyperactivity disorder” OR ADHD) AND TS=(communication OR language OR speech OR pragmatics OR “auditory processing”) AND TS=(diet OR nutrition OR micronutrients OR omega-3 OR microbiome OR “gut-brain axis” OR “oxidative stress” OR inflammation)
Google Scholar	ADHD AND communication AND (diet OR nutrition OR microbiome OR “oxidative stress”)
